# Electrochemical Performance of Niobium MXenes with Lanthanum

**DOI:** 10.1021/acsami.4c10354

**Published:** 2024-09-16

**Authors:** Meriene Gandara, Bianca Fortes Palley, Lazar Rakočević, Dušan Mladenović, Ana Popović-Bijelić, Biljana Šljukić, Emerson Sarmento Gonçalves

**Affiliations:** † Technological Institute of Aviation, Space Science and Technology Graduate Program, Praça Marechal Eduardo Gomes, 50, 12228-900 São José dos Campos, Brazil; ‡ Vinča Institute of Nuclear Sciences, Department of Atomic Physics, 12-14 Mike Petrovića Street, 11351 Belgrade, Serbia; § University of Belgrade, Faculty of Physical Chemistry, Studentski trg 12-16, 11158 Belgrade, Serbia; ∥ Center of Physics and Engineering of Advanced Materials, Laboratory for Physics of Materials and Emerging Technologies, Chemical Engineering Department, Instituto Superior Técnico, Universidade de Lisboa, 1049-001 Lisbon, Portugal; ⊥ Institute of Aeronautics and Space, Divisão de Materiais, Praça Marechal Eduardo Gomes, 50, 12228-904 São José dos Campos, Brazil

**Keywords:** 2D materials, MXene, niobium MXene, niobium−lanthanum MXene, energy storage, microsupercapacitors

## Abstract

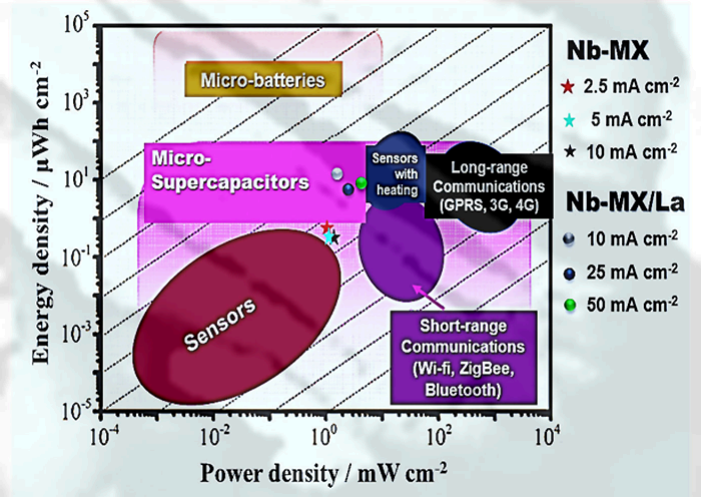

MXenes are the newest class of two-dimensional nanomaterials characterized by large surface area, high conductivity, and hydrophilicity. To further improve their performance for use in energy storage devices, heteroatoms or functional groups can be inserted into the Mxenes’ structure increasing their stability. This work proposes insertion of lanthanum atoms into niobium-MXene (Nb-MX/La) that was characterized in terms of morphogy, structure, and electrochemical behavior. The addition of La to the Nb-MXene structure was essential to increase the spacing between the layers, improving the interaction with the electrolyte and enabling charge/discharge cycling in a higher potential window and at higher current densities. Nb-MX/La achieved a specific capacitance of up to 157 mF cm^–2^, a specific capacity of 42 mAh cm^–2^ at 250 mV s^–1^, a specific power of 37.5 mW cm^–2^, and a specific energy of 14.1 mWh cm^–2^ after 1000 charge/discharge cycles at 50 mA cm^–2^.

## 1 Introduction

To meet the energy demands of advanced technology in electroelectronic devices, wearable electronics, self-powered wireless networks, sensors, and IoT electronics, electrochemical energy storage devices such as microsupercapacitors (MSCs) are being designed. These devices are capable of storing charge and dissipating a high power in a short discharge time with a long cycling capacity. To maintain electrochemical performance and stability, MSC electrodes must be composed of materials with high charge transport and storage capacity.^1,2^

MXene is a recently discovered class of two-dimensional (2D) materials, first reported in 2011, that is widely used for various applications, particularly for electrochemical energy storage. These materiala have a general formula M_n+1_X_n_T_*x*_, and they are composed of a transition metal M, carbon and/or nitrogen X, with n = 1–4, and surface functional groups (O, OH, F, and Cl) T_*x*_. They can be synthesized by etching the corresponding precursor, the MAX phase.^3^

MXenes have properties that make them promising for use as materials for MSC electrodes, including a large surface area and high conductivity that favor the process of ionic diffusion and rapid electronic transport, as well as hydrophilicity that makes them compatible with aqueous electrolytes and thermal stability. The storage mechanism of MXenes is complex and depends mainly on their different structures, surface functional groups, precursor, and synthesis, among other factors.^4^

Zhang et al. prepared MXene (Ti_3_C_2_T_*x*_) inks for extrusion and inkjet printing, which were used in microsupercapacitor devices with high-performance volumetric capacitance and energy density.^5^ Li et al. proposed a flexible asymmetric microsupercapacitor (Ti_3_C_2_T_*x*_//polypyrrole (PPy)/MnO_2_) with MXene ink deposited on a graphite paper as the negative electrode, exhibiting a maximum areal capacitance and energy density of 61.5 mF cm^–2^ and 6.73 μWh cm^–2^, respectively.^6^ Zhu et al. proposed a new high-concentration (18 mol kg^–1^) gel electrolyte, ’water in LiBr’ (WiB), for symmetric MXene MSCs to achieve high energy and overcome the challenge of low voltage windows in aqueous electrolytes (typically ≤0.6 V); a potential window of 1.8 V and high energy density were indeed achieved.^7^

Although MXenes show promise for applications with extensive advantages, they are susceptible to degradation of their electrochemical performance due to restacking of their layers and vulnerability to oxidation when exposed to air and aqueous media.^8^ The introduction of heteroatoms into 2D materials has proven to be a successful strategy for adjusting their physicochemical properties.^9^ For direct application in energy storage devices, the advantages of inserting heteroatoms into the structure of MXenes consist of adjusting the interlayer structure, reducing charge-transfer resistance, and increasing conductivity, increasing stability and electrochemical performance.^10^

Therefore, this work proposes the insertion of the rare earth element lanthanum (La) into niobium (Nb) MXene to enhance its electrochemical properties and performance as an active material for MSC energy storage devices.

## 2 Experimental Section

### 2.1 Obtaining MAX PHASE Precursor of MXene

The MAX phase was prepared by mixing equimolar (1,1:1) amounts of Nb (99.4%, 325 mesh ∼44 μm, CBMM), Al (99.5%, 9.5 μm, ALCOA Company), and NbC (99.7%, 325 mesh ∼44 μm, CBMM) in a planetary mill for 1 h. The powdered reagents were uniaxially pressed at 55 MPa to a pastel of 15 mm in diameter and 3 mm in height. The reaction took place in a tube furnace (Spembly) in a graphite crucible in an argon atmosphere for 3 h at 1650 °C with temperature increasing rate of 17 °C min^–1^. Subsequently, the obtained MAX phase pastille was crushed and sieved to 400 mesh to prepare the Nb-MXene.

### 2.2 Obtaining Niobium Carbide MXenes (Nb-MX) and Lanthanum-Doped Niobium Carbide MXenes (Nb-MX/La)

The MILD method was used to obtain Nb-MX multilayers: 1 g of Nb MAX phase, 1 g of LiF (Sigma-Aldrich), and 8.5 mL of HCl (37%, Neon) were stirred at 50 °C for 96 h. After the reaction, the material was washed with DI water to pH ∼ 6, centrifuged at 2750 rpm for 5 min, and dried in an oven at 120 °C for 4 h.

The insertion of La into Nb-MX was carried out by mixing 400 mg of multilayer Nb-MX powder in 50 mL of a 1 M La(NO_3_)_3_ solution under stirring at 80 °C for 4 h C. The material was then washed with DI water until pH ∼ 6 and centrifuged at 2750 rpm for 5 min. The precipitate was dried in an oven at 120 °C for 4 h to obtain multilayer Nb-MXene with La powder (Nb-MX/La).

### 2.3 Conductive Ink and Electrode Preparation

Conductive Nb-MX and Nb-MX/La inks were prepared without a binder at a concentration of 10 mg mL^–1^. The materials were dispersed in DI water by vigorous stirring in a magnetic stirrer for 24 h, followed by sonication in an ultrasonic bath for 1 h.

The current collector used for the electrochemical tests was glassy carbon, on which the inks were deposited by drop-casting and drying at room temperature, forming a film of 0.0706 cm^–2^ area at a mass loading of 2.63 mg cm^–2^.

The same process of preparing the conductive ink was carried out with Nb-MX and Nb-MX/La, which were also tested electrochemically in order to compare their performance.

### 2.4 Physicochemical Characterization of MAX Phases and Nb-MX

The materials were characterized by X-ray diffraction (XRD) analysis using Ultima IV - Rigaku equipment, with Cu Kα radiation (λ = 1.54056 Å), Ni filter, 40 kV and 30 mA with a 0.02° scanning step. The morphology was analyzed by scanning electron microscopy using a field emission gun (FEG-SEM) with elemental analysis by energy dispersive spectroscopy (EDS) using Tescan Mira 3LM equipment.

X-ray photoelectron spectroscopy (XPS) was performed by SPECS Systems with an XP50 M X-ray source for a Focus 500 X-ray monochromator and PHOIBOS 100/150 analyzer. Source used was Al Kα (1486.74 eV) at a 12.5 kV and 32 mA. Spectra of La 3d, F 1s, and Nb 3d were recorded at a constant pass energy of 20 eV, step size of 0.1 eV, and dwell time of 2 s in the FAT mode. All positions were referred to C 1s at 284.8 eV.

Electron paramagnetic resonance (EPR) spectroscopy was performed at 295 K on a Bruker Biospin Elexsys II E540 EPR spectrometer. The samples in powder form (∼10–15 mg) were placed into 1 mm-diameter gas-permeable Teflon tubes (Zeus Industries Inc., Largo, FL, USA), and inserted into a quartz EPR cuvette (inner diameter 3 mm, Wilmad LabGlass, Vineland, NJ, USA) for measurements. The experimental parameters were as follows: microwave frequency 9.85 GHz, microwave power 10 mW, modulation amplitude 1 G, and modulation frequency 100 kHz.

### 2.5 Electrochemical Performance

Electrochemical analyses were carried out by cyclic voltammetry (CV) from 0 to 1 V vs RHE for Nb-MX and from 0 to 1.5 V vs RHE for Nb-MX/La at different scan rates (5, 10, 20, 50, 75, 100, 200, and 250 mV s^–1^). Moreover, galvanostatic charge and discharge at current densities of 2.5, 5, 10, 25, and 50 mA cm^–2^ for 1000 cycles were carried out in the same potential windows as the CV. Finally, electrochemical impedance spectroscopy (EIS) was carried out at the open circuit potential (OCP) in the frequency range from 10 mHz to 1 MHz, before and after GCD. All the electrochemical analyses were carried out using an IVUIUM potentiostat/galvanostat in 3-electrode cells, with the working electrode made of conductive Nb-MX and Nb-MX/La paint deposited on a glassy carbon, the counter electrode made of platinum and the saturated calomel reference electrode in 1 M H_2_SO_4_ electrolyte. All measured potentials were converted to a reversible hydrogen electrode (RHE) scale.

Equations 1 and 2 describing the specific capacitance *C*_*s*_(11) and specific capacity *C*_*ps*_(12) were used to calculate their values from the cyclic voltammetry data:
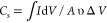
1
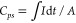
2where *I is* current, Δ*V* the potential window, A geometric active area of the electrode, and υ is the scan rate.

Eqs 3, 4 and 5 for Coulombic efficiency *η,*(13) specific energy *E*_*s*_ and specific power *P*_*s*_,^14^ respectively, were used to evaluate GDC performance:
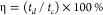
3
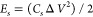
4
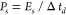
5where *t*_*d*_ is the discharge time, *t*_*c*_ charge time, and Δ*t*_*d*_ discharge time variation.

Using EIS analysis, the complex capacitance can be associated with phenomena occurring in the micro- or supercapacitor cell. *C*’(ω), the real part, is associated with the capacitance of the device during the discharge process at low frequencies and *C*″(ω), the imaginary part, corresponds to the energy dissipated from the entire system by irreversible loss. The complex capacitance can be represented by eqs 6, 7 and 8:^15,16^
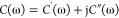
6
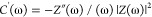
7
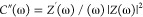
8where *Z*’(ω) is the real impedance, and −*Z*″(ω) is the imaginary impedance.

Equation 9 represents the relaxation time constant (τ), the inverse of the frequency *f*, related to the minimum time required for all the charge to be released in the energy storage device with an efficiency greater than 50%^16^
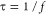
9

## 3 Results and Discussion

### 3.1 Physicochemical Characterization

**Figure 1:**
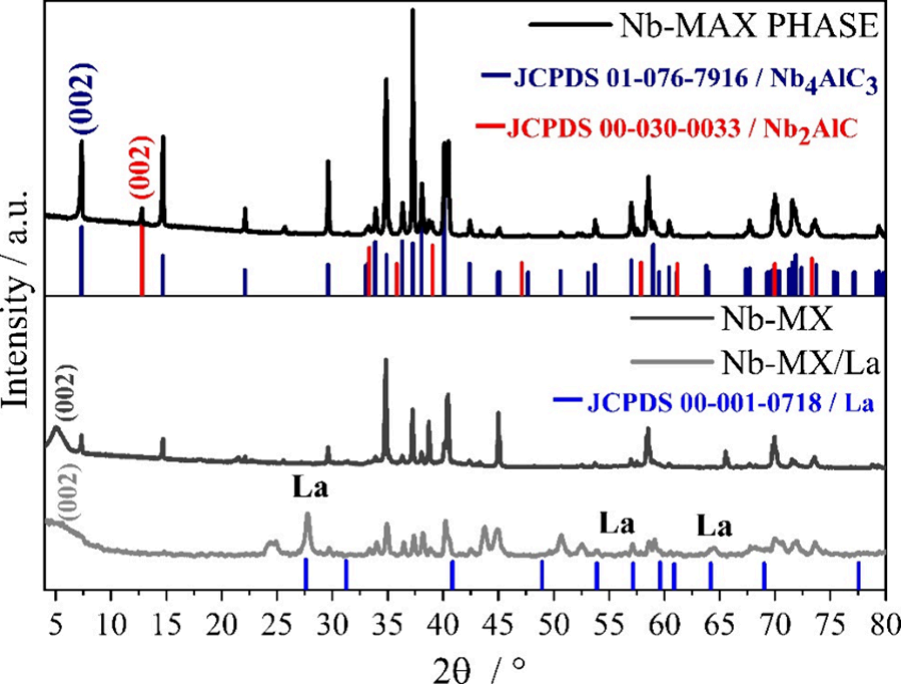
Comparison of the diffractograms of the MAX phase and the respective JCPDS cards (01–076–7916 and 00–030–0033) and comparison of diffractograms of the MAX phase, MXene without added lanthanum (Nb-MX), and MXene with lanthanum (Nb-MX/La) along with the JCPDS (00–001–0718) card.

The MAX phase precursor of niobium carbide MXene (Nb-MX) consists of a mixture of Nb_4_AlC_3_ and Nb_2_AlC phases, as demonstrated by the formation of characteristic plane (002) with reflections observed at 2θ of 7.3° and 12.7° (002), respectively (Figure 1).^17,18^ Naturally, the formed MXene may be composed of Nb_4_C_3_T_*x*_ and Nb_2_CT_*x*_ with different properties for each phase. Since the properties of the phases are commonly studied separately, this work proposes the study of the properties of the material formed by the mixture of phases.

The success of the etching process and formation of MXene can be observed through the reduction in peak intensity, the difference in layer spacing (*d-*spacing), and particularly the shift of the (002) plane (Figure 1).^19^ The *c*-LP lattice parameter and *d*-spacing values were calculated using the Bragg and hexagonal phase structure equations.^20−22^Table 1 shows that the *d*-spacing values for Nb-MX and Nb-MX/La have increased, indicating the opening of the nanolayers of the MAX phase. Additionally, they are comparable to the values obtained for Nb_4_C_3_T_*x*_-6 (1.71 nm) and Nb_4_C_3_T_*x*_-8 (1.78 nm) in the work of Zhao et al.^23^

**Table 1:** Comparison of 2θ Corresponding to the Reflection from the (002) Plane, *c*-LP Lattice Parameter, and *d*-Spacing of MAX Phase, Nb-MX, and Nb-MX/La

	2θ/° (002)	*c*-LP/nm	*d-*spacing/nm
Nb_4_AlC_3_	7.3	2.39	1.19
Nb_2_AlC	12.7	1.38	0.69
Nb-MX	5.1	3.45	1.72
Nb-MX/La	4.9	3.59	1.79

Metallic elements can be incorporated into MXenes by simple dispersions of MXene in saline solutions or by chemical reactions;^24^ when mixed or reacted in high concentrations, significant oxidation of MXene and neutralization of the negative surface charge can occur.^25^

A decrease in the *c*-LP network parameter can be indicative of two effects: a simple ion exchange between compounds and the MXene surface or a strong electrostatic interaction. The strong electrostatic interaction of La^3+^ with negative charges on the MXene surface can decrease the spacing between layers (reducing the *c*-LP value) further causing agglomeration of the MXene sheets, as observed in the work of Iqbal et al.^26^

However, the agglomeration of the Nb-MX/La sheets did not occur, as the *c-*LP did not decrease; this might be an indication that there is not just a simple interaction phenomenon between La^3+^ and the MXene surface. Conversly, when La was herein added to Nb-MX, forming Nb-MX/La, an increase in the *d*-spacing and *c*-LP lattice parameter is observed. According to Zhang et al., the crystalline material with La^3+^ ions can lead to an expansion of the lattice.^27^ The ionic radius of La^3+^ (0.103 nm) is larger than that of Nb^2+^ (0.08 nm), and the increase in the *c* lattice parameter may indicates the occurrence of an interstitial incorporation in the structure.^28,29^ MXenes with added heteroatoms increase the surface area and *c*-LP lattice parameter by introducing defects in the structure, which contributes to improving their specific properties in energy storage device applications.^29^

Moreover, the addition of desired metals to MXene, through reactions with salts of these metals, it is possible to form crystals.^25^ Maxima at 2θ = 27.73°, 53.78°, 57.13° and 64.44° belong to La compounds (JCPDS 00–001–0718)^27^ indicating that there may be an excess of La in MXene, according to the peak shifts (2θ= 24.64°, 34.97°, 40.65°, 73.75°).^26^ Subsequently, it can be observed from the elemental distribution (Figure 3) that there is La on the surface of MXene and in clusters together with elements such as F and O (see below for more details). The La compounds are confirmed by the XPS analysis (Figure 4) which shows bonds between La–O and La–F, forming compounds of La_2_O_3_ and LaF_3_, in addition to the expected bonds between La–Nb that are also evident (see below for more details).

**Figure 2:**
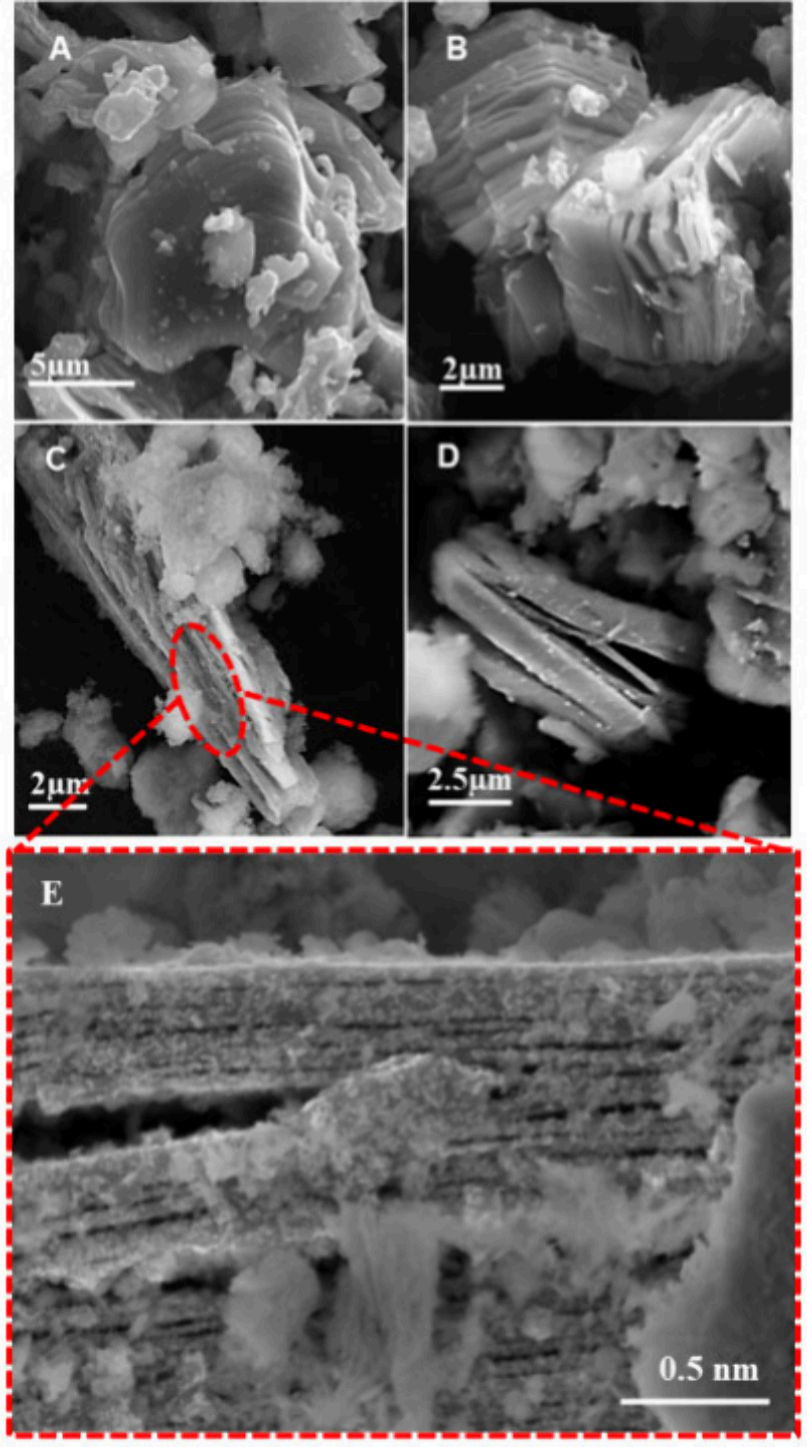
(A) Typical nanolayed morphology of the MAX phase, (B) morphology of Nb-MX indicating success in the etching process, (C, D) morphology of MXenes with La, Nb-MX/La, and (E) higher magnification of Nb-MXene/La showing the difference in morphology on the surfaces of the MXene layers in the presence of La.

The typical morphology in closed nanolayers for the MAX phase and open for MXenes can be observed in Figure 2. The opening of the layers is also an indication of successful etching of the MAX phase for the formation of MXene.^21^Figure 2C and 2-D show a different morphology of La MXene flake. As can be seen in Figure 2E, the surface morphology of the MXene layers changes in the presence of La. At higher magnification, it is possible to observe the roughness of the surface of the Nb-MX/La layers. This change in morphology is an indication that La has bonded with the MXene, not only the formation of La salt crystals on its surface, that assume a different morphology. In the work of Yang et al., the same effect of morphology change with roughness of the MXene film occurs with the incorporation of Ni in its structure.^25^

**Figure 3:**
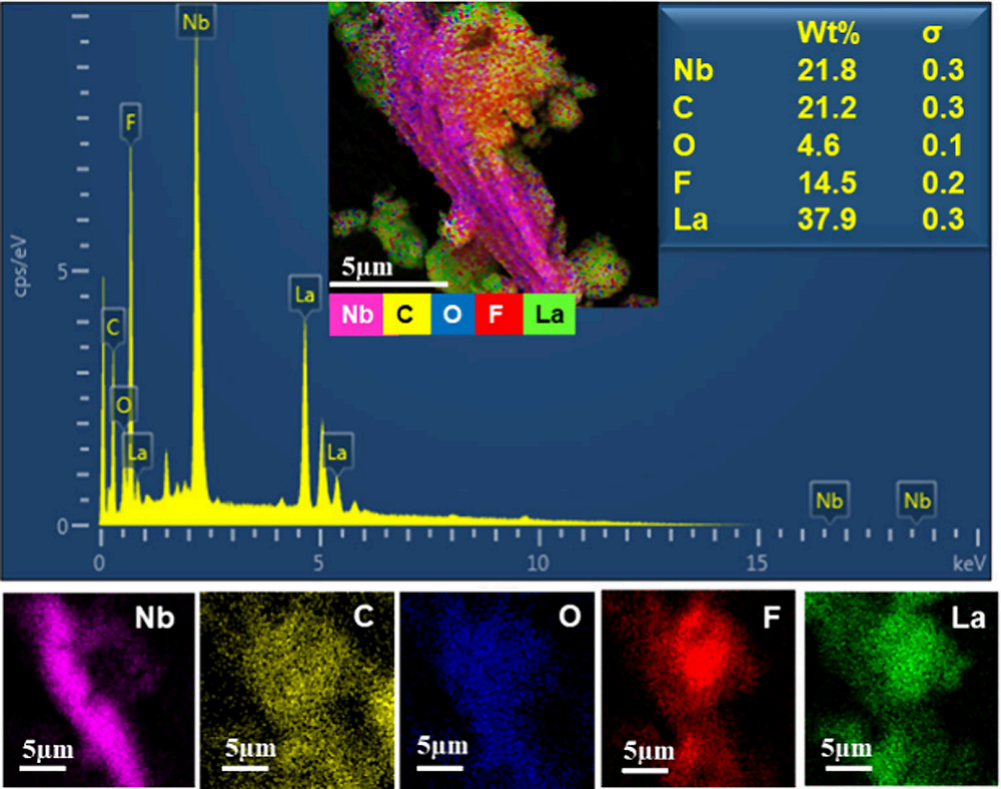
Elemental analysis by EDS of Nb-MX/La highlights the composition by chemical element related to the morphology of the material.

Elemental analysis by EDS reveals that Nb-MXene/La is composed of the surface functional groups T_*x*_ of MXene (-O, -F). The excess of La is evidenced by its high wt %, likely due to its high concentration in relation to MXene during the synthesis process (Figure 3).

**Figure 4:**
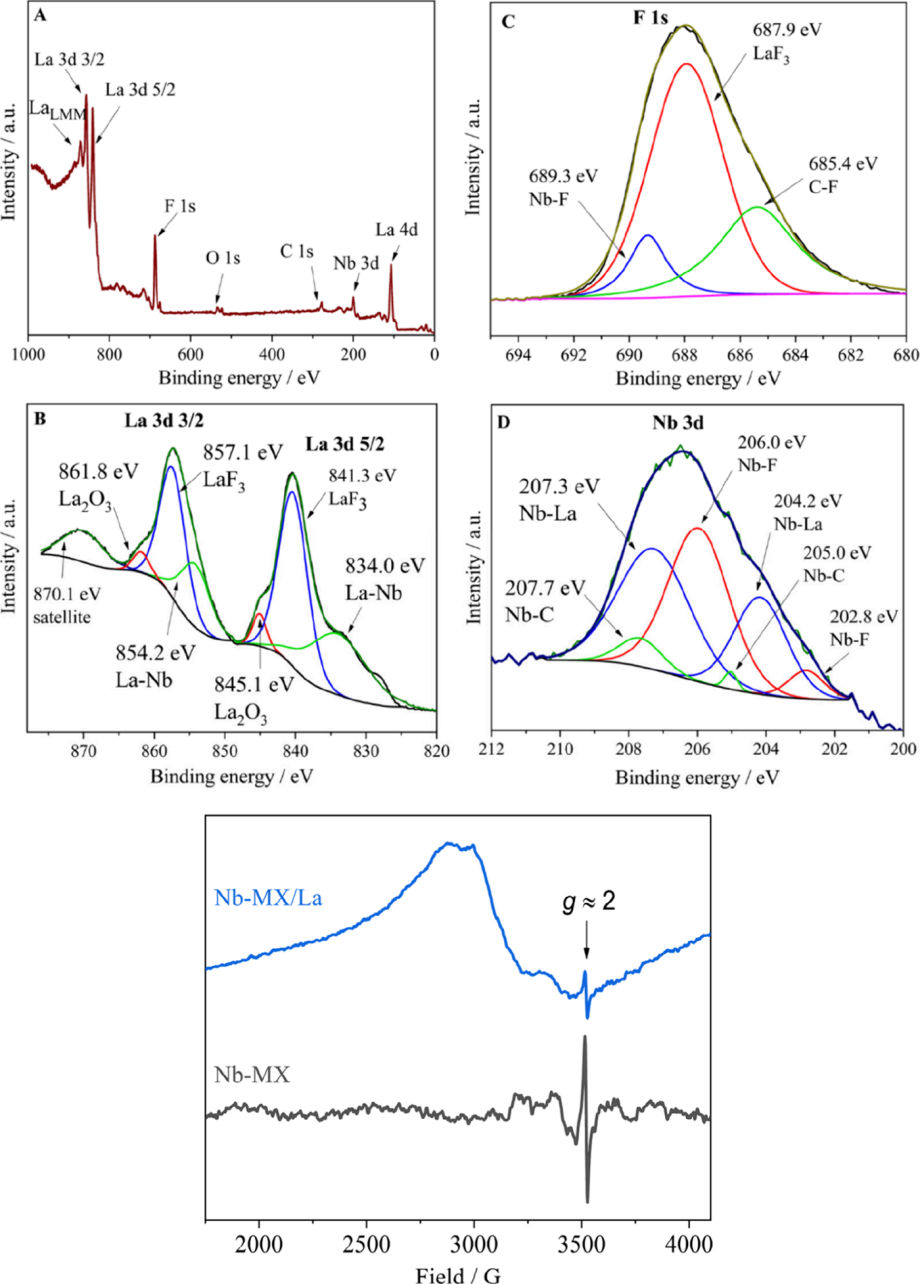
XPS spectra of Nb-MX/La: (A) survey, (B) La 3d, (C) F1s, (D) Nb 3d and (E) X-band EPR spectra Nb-MX (black line) and Nb-MX/La (blue line) measured at 295 K.

XPS analysis was conducted to evaluate the chemical bonds in MXene after the La insertion process (Figure 4). Survey XPS spectrum (Figure 4A) shows the presence of La, F, O, C, and Nb.

The high resoultion spectrum of La 3d (Figure 4B) shows a characteristic doublet consisting of La 3d 3/2 and La 3d 5/2. Each is deconvoluted into three components. Peaks at 857.1 and 841.3 eV (52.8% of total La 3d emission) can be assigned to a layer of LaF_3_ that covers the surface of MXene.^30^ Since the XPS is a strictly surface technique, this layer that covers the surface reduces the signal coming from the MXene, making Nb, C and O signals much weaker than expected. Small peaks at 861.8 and 845.1 eV (4.9% of La 3d emission) can be assigned to remaining La on the surface that formed the oxide La_2_O_3_.^31^ Peaks at 854.2 and 834.0 eV (34.1% of emission) are attributed to La–Nb bond that lanthanum formes with the MXene.^32^ Final remaining peak at 870.1 eV (8.2%) is a satellite feature originating from La_LMM_ Auger line.^30−32^

The high resolution spectrum of F 1s (Figure 4C) is deconvoluted into three components. The peak at 687.9 eV (61.7% of total F 1s emission) that accounts for the majority of F present can be attributed to LaF_3_, confirming its presence on the surface of MXene.^30^ The peak at 689.3 eV (9.9% of F 1s emission) is assigned to the Nb–F bond, indicating that F also binds to the surface of MXene. The peak at 685.4 eV (28.4% of emission) is attributed to the C–F bond that F forms with the MXene.^33^

The high resonance spectrum of Nb 3d (Figure 4D) consists of Nb 3d 3/2 and Nb 3d 5/2 doublets that are deconvoluted into three components each. Peaks at 206.0 and 202.8 eV (40.4% of total Nb 3d emission) are assigned to Nb–F bond confirming that F binds to the surface of MXene.^33^ Peaks at 207.3 and 205.0 eV (54.0% of Nb 3d emission) correspond to Nb–La bond between La and MXene.^32^ Peaks at 207.7 and 205.0 eV (5.6% of emission) originate from Nb–C bonds in the structure of the MXene.^23,34^ As mentioned above, the signal from bonds originating from the structure of MXene appears weaker than what would be expected otherwise due to LaF_3_ covering the surface of MXene.

La formed bonds with Nb as well as with the functional groups of O and F, as previously observed in Figure 3. The excess La may have formed an additional layer of La_2_O_3_ and LaF_3_ on the MXene surface in addition to the expected insertion process. Another phenomenon that can promote the formation of La_2_O_3_ and LaF_3_ is the occurrence of ion exchange between La^3+^ ions and surface T_*x*_ groups (-O, -F) with negative charge.^35^

The insertion of La into MXenes can cause disturbances and defects in the distribution of electrons, forming active sites. The contribution of La atoms has a greater impact on the material’s surface, which can enhance its electronic properties.^36^ Bae et al. conducted density functional theory (DFT) calculations to study the electronic structures of different MXenes.^37^ By considering the oxidation state and orbital distribution (number of electrons belonging to the transition metal), it is possible to infer properties related to the electrical behavior of MXenes. For compounds with a d^0^ configuration (empty d band), the electrical behavior tends to be more insulating, while for compounds with dn configuration, where *n* > 0 is a partially filled even-numbered layer, the MXene assumes metallic character.

DFT calculations predict that metals with a higher energy level in *d* orbitals (such as La) modify the intrinsic properties of MXene, such as magnetism, layer thickness, and lattice parameters (observed in section 3.1, Table 1). When a metal is bonded to the M layer of MXene (seen in Figure 4B, D), the hybridization of the electronic state of the d orbital layers increases the electron density around the Fermi level due to interactions between the orbitals. This phenomenon causes apparent changes in the electronic properties of the material, increasing its metallic character, directly influencing the electrochemical processes.^26^

**Figure 5:**
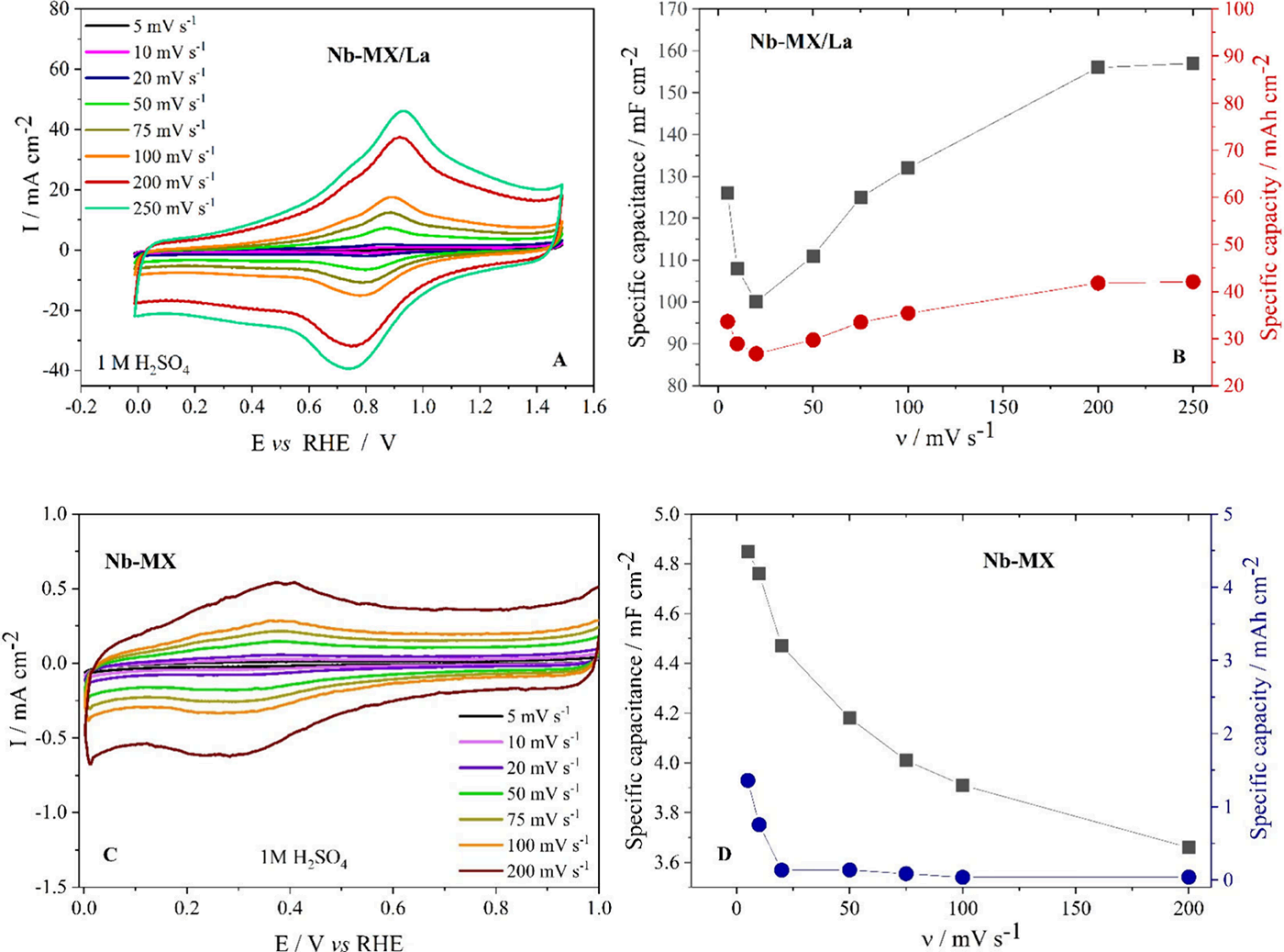
Cyclic voltammetry profile of (A) Nb-MX/La and (C) Nb-MX in 1 M H_2_SO_4_ at different scan rates (5.10, 20, 50, 100, 200, 250 mV s^–1^). Specific capacitance and specific capacity of (B) Nb-MX/La and (D) Nb-MX at different scan rates.

The EPR spectra of both Nb-MX and Nb-MX/La recorded at 295 K (Figure 4E) display a narrow signal (line width of 4.8 G) centered at *g* ≈ 2, characteristic of polarons,^38−40^ indicating the presence of this type of electronic charge carriers in MXenes. Additionally, the spectrum of Nb-MX/La also contains a very broad signal (∼400 G) which can be speculated to originate from paramagnetic Nb^4+^ ions created by the oxidation of MXene upon insertion of La. The normalized intensities of the polaron-attributed EPR signals, calculated per mg of sample, are 0.058 for Nb-MX, and 0.017 for Nb-MX/La. This shows that Nb-MX/La, which demonstrated greater current density and charge storage compared to the MXene without La (Figure 5), contains ca. 3x lower concentration of the polaronic species. The observed inverse correlation between polaron concentration and capacitance/conductivity has previously been reported for PANI,^39^ suggesting that the mobility and distribution of polarons, and not only their concentration, may significantly influence electrical properties.^41^ Moreover, it has been proposed that at higher doping levels of PANI salts, the concentration of polarons decreases due to the formation of bipolarons, which are not spin carriers, although both species act as charge carriers.^40^ Therefore, it may be possible that in Nb-MX/La, the effective concentration of bipolarons is higher compared to that in Nb-MX. Most likely, *in situ* EPR studies coupled with CV experiments could provide detailed information about possible polaron concentration changes at different potentials.

The positions of the T_*x*_ groups also influence the stability and electronic structure. MXene theoretical La_2_CO_2_ are attributed to the d^0^ configuration, making them more insulating than those terminated in La_2_CF_2_ with partially filled orbitals.^37^ The Nb-MX/La obtained herein presents a higher proportion of fluorinated groups than oxygen-terminated functional groups, as shown in Figures 3 and 4B–D. This could potentially enhance the material’s electronic performance.

### 3.2 Electrochemical Performance by Cyclic Voltammetry (CV)

Cyclic voltammetry is a widely used technique to investigate the electrochemical behavior of a system, particularly to evaluate charge storage mechanisms in electrode materials. The shape of the curves can indicate whether the behavior is rectangular, indicating an electric double layer capacitor (EDLC), or whether there are peaks of not very pronounced redox pairs, indicating pseudocapacitance (PC), or more pronounced peaks, indicating complete faradaic behavior.^42,43^ The CV profiles of MXene with and without La clearly differ. Nb-MX/La exhibits a more pronounced redox couple around 0.7 V, while Nb-MX shows a peak around 0.4 V. (Figuras 5-A, C).

The electrochemical performance (specific capacitance and capacity) of materials for charge storage can be calculated by integrating CV curves.^43^ It is important to note that materials with the contribution of charge storage by EDLC effects and simultaneous diffusion of ions are more complex due to the physicochemical interactions of charges in addition to the double layer and the irregular or porous geometry of the surface. Therefore, the capacity must be calculated considering all charge storage effects, while the capacitance will be a measure of apparent charges.^12,44^

Lanthanum, by modifying the chemical structure of MXene, favored greater current density and charge storage, increasing the specific capacitance by 2 orders of magnitude and the specific capacity more than 10 times in relation to MXene without La. The presence of La in Nb-MX/La also modified the charge storage profile throughout the scan rates, increasing up to 200 mV s^–1^ and remaining constant at 250 mV s^–1^, while for Nb-MX there was a decrease in charges from 5 mV s^–1^ to 200 mV s^–1^ (Figure 2B, D). Moreover, with the presence of La (Nb-MX/La), the material supported a greater potential variation of 1.5 V in relation to 1 V in the case of Nb-MX, without presenting evident parallel reactions characteristic of redox couples in water electrolysis. In the work of Li et al., similar behavior was demonstrated by Nb MXene in an aqueous medium; Nb_2_CT_*x*_ changed from capacitive to faradaic behavior with diffuse control when the potential window was increased from 2 to 2.4 V.^45^

The specific capacitance of Nb-MX/La at 20 mV s^–1^ was 100 mF cm^–2^, higher than that of titanium carbide MXene (Ti_3_C_2_T_*x*_) with 23.6 mF cm^–2^, under the same conditions for the same application, in work Huang and Wu.^46^ MXene aerogel with reduced graphene oxide described in the work of Yue et al. exhibited a specific capacitance of 34.6 mF cm^–2^ at 1 mV s^–1^. However, its performance was inferior compared to Nb-MX/La at a low scan rate (126 mF cm^–2^ at 5 mV s^–1^).^47^ Wang et al. prepared an ink using MXene (Ti_3_C_2_T_*x*_)/sodium alginate-Fe^2+^ and tested it in gel electrolyte (PVA/H_2_SO_4_), delivering 123.8 mF cm^–2^ at 5 mV s^–1^, a value similar to that of Nb-MX/La.^48^

MXenes, like other 2D materials, tend to reduce their interlayer spacing due to strong Van Der Waals forces and hydrogen bonding of their functional groups. This reduces the space between the nanosheets, hindering interaction with electrolyte ions and the intercalation/deintercalation process.^49^ However, with the insertion of La into the structure, the spacing between the layers increased, as discussed in section 3.1. This increased spacing facilitates ion diffusion and reduces the kinetic barrier of the redox reaction between the electrolyte and the material‘s surface, improving charge transport and storage.^50^ La may have acted as a nanospacer between the MXene nanosheets and not allowed the agglomeration of the MXene layers, as there was no decrease in c-LP (section 3.1), consequently improving its electrochemical performance.

To better evaluate electrochemical phenomena and charge storage kinetics in the material, one can estimate the contributions of internal diffusion-controlled storage and external surface-controlled contributions through cyclic voltammetry, as shown in eq 10:
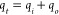
10Given that *q*_*t*_ is the total charge, *q*_*i*_ is the charge on the internal surface, and *q*_*o*_ is the charge on the external surface, the total charges stored with respect to the mechanisms of diffusion-control and pseudocapacitance can be described in eq 11:
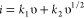
11in which *k*_*1*_*υ* represents the pseudocapacitive contribution and *k*_*2*_*υ*^*1/2*^ represents the diffusion contribution t the current. Therefore, the relationship between the peak current obtained at a certain scan rate, using empirical data that allows for a rapid kinetic evaluation of the material, is described by eq 12:
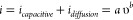
12where *a* and *b* are adjustable parameters of the equation and the value of *b* can be calculated from the slope of log(*i*) versus log(υ). A value of *b* of 0.5 indicates diffusion-controlled charge storage processes (faradaic processes), while a value of 1 is characteristic of surface behavior in pseudocapacitive processes.^51,52^

**Figure 6:**
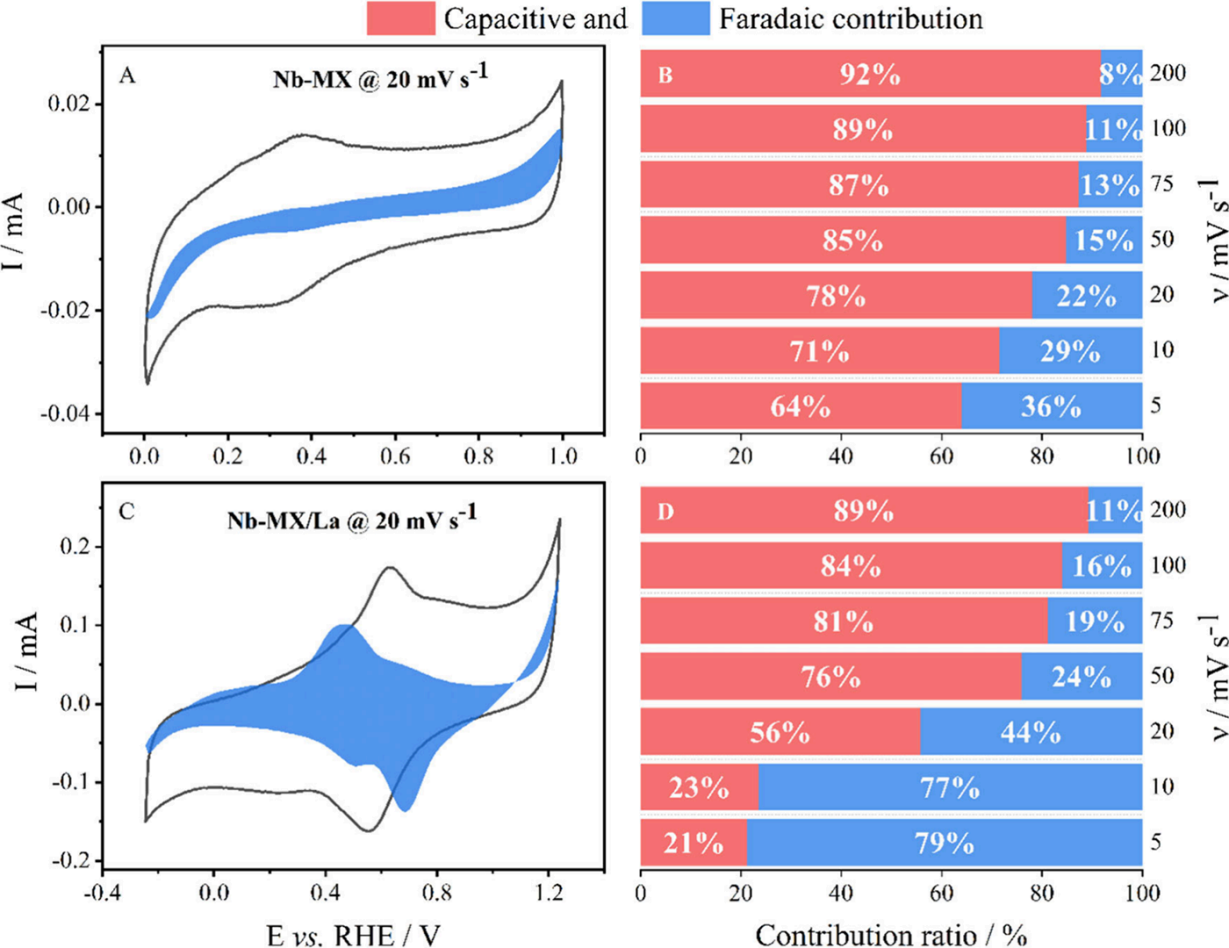
Analysis of charge storage contribution mechanisms by cyclic voltammetry: CV of A- Nb-MX and C- Nb-MX/La in 1 M H_2_SO_4_ at a scan rate of 20 mV s^–1^, along with the percentage contribution of capacitive and faradaic phenomena for B- Nb-MX and D- Nb-MX/La at different scan rates.

Figure 6 can be used to distinguish between charge storage mechanisms in Nb-MX compared to Nb-MX/La. At lower scan rates, where the process of charge accommodation in the material is better defined, a greater contribution of controlled diffusion (Faradaic process) is noticeable in both MXenes. This behavior is similar to that observed in the MXene studied by Guan et al., but with greater intensity for Nb-MX/La.^53^ Ions can be intercalated within the layers of MXenes or adsorbed onto their terminal groups, where the process of charge transfer occurs depending on the degree of hybridization between their orbitals.^54^ The addition of La as an extra functional group in the structure of MXene not only increases the layer spacing but may also cause network distortions, increase the presence of charge carriers, and enhance its redox capacity, thereby amplifying the faradaic effects.^41^

### 3.3 Electrochemical Performance by Galvanostatic Charge and Discharge (GCD)

The electrochemical tests were carried out mainly to evaluate the material properties for future applications in energy storage devices. Thus, with the aim of monitoring the stability of the material in the charge and discharge process in the first cycles. And compare whether the insertion of a heteroatom in MXene influences the charge storage capacity.

**Figure 7:**
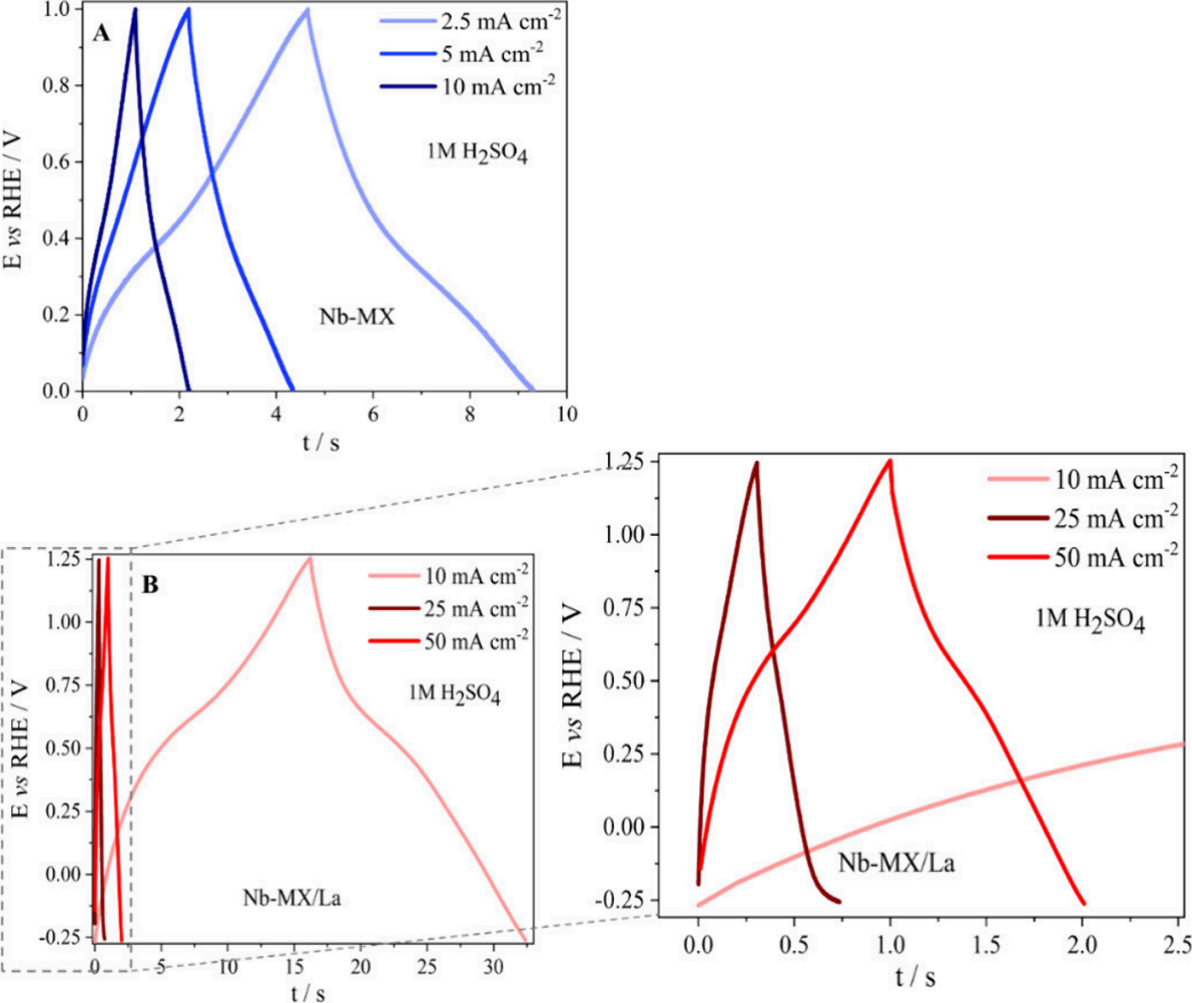
GCD analysis in 1 M H_2_SO_4_ for (A) Nb-MX and (B) Nb-MX/La at different current densities (2.5, 5, and 10 mA cm^–2^),.

**Figure 8:**
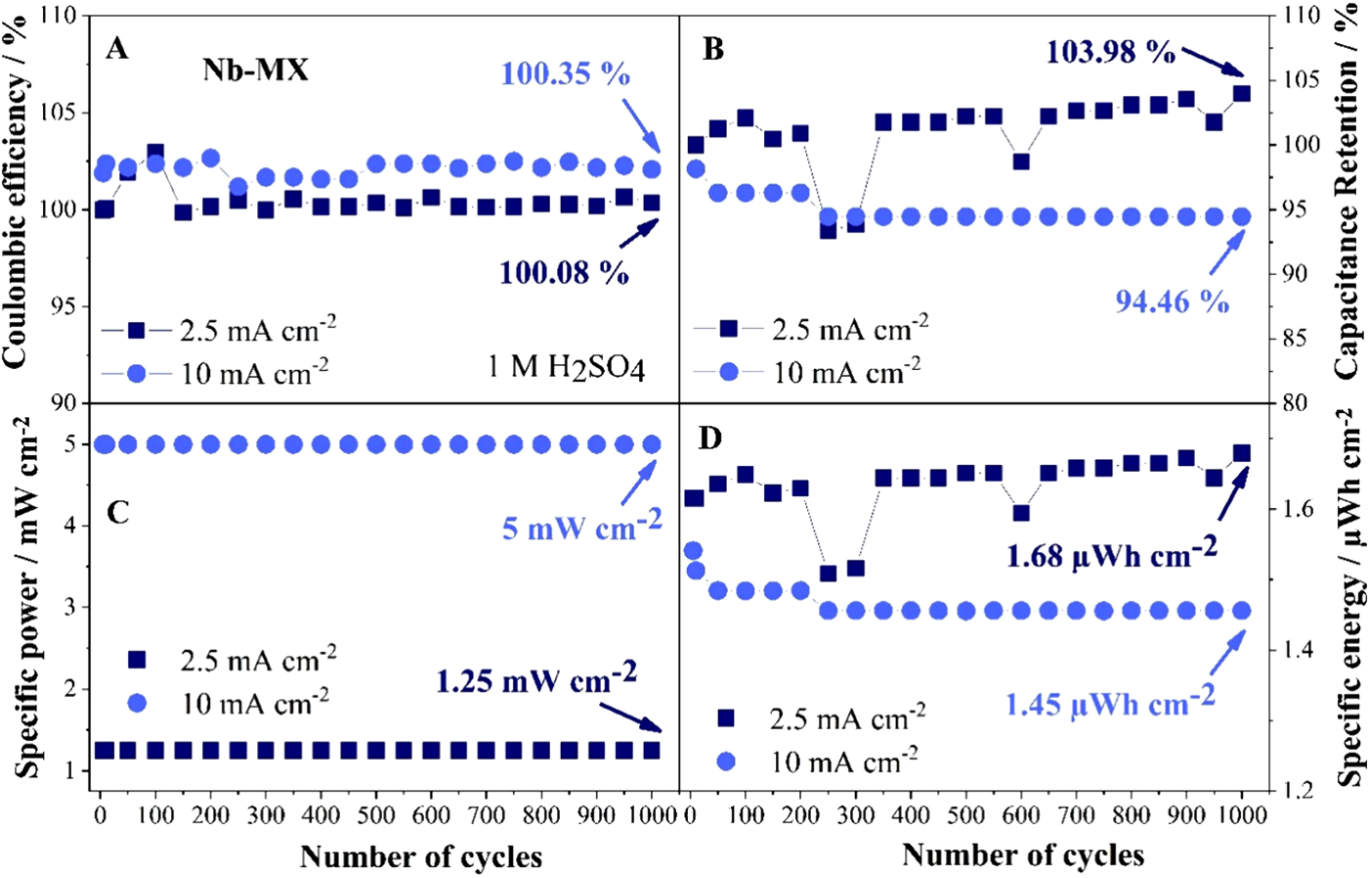
GCD performance of Nb-MX in 1 M H_2_SO_4_: A- Coulombic efficiency, B- capacitance retention, C- specific power, and D- specific energy.

**Figure 9:**
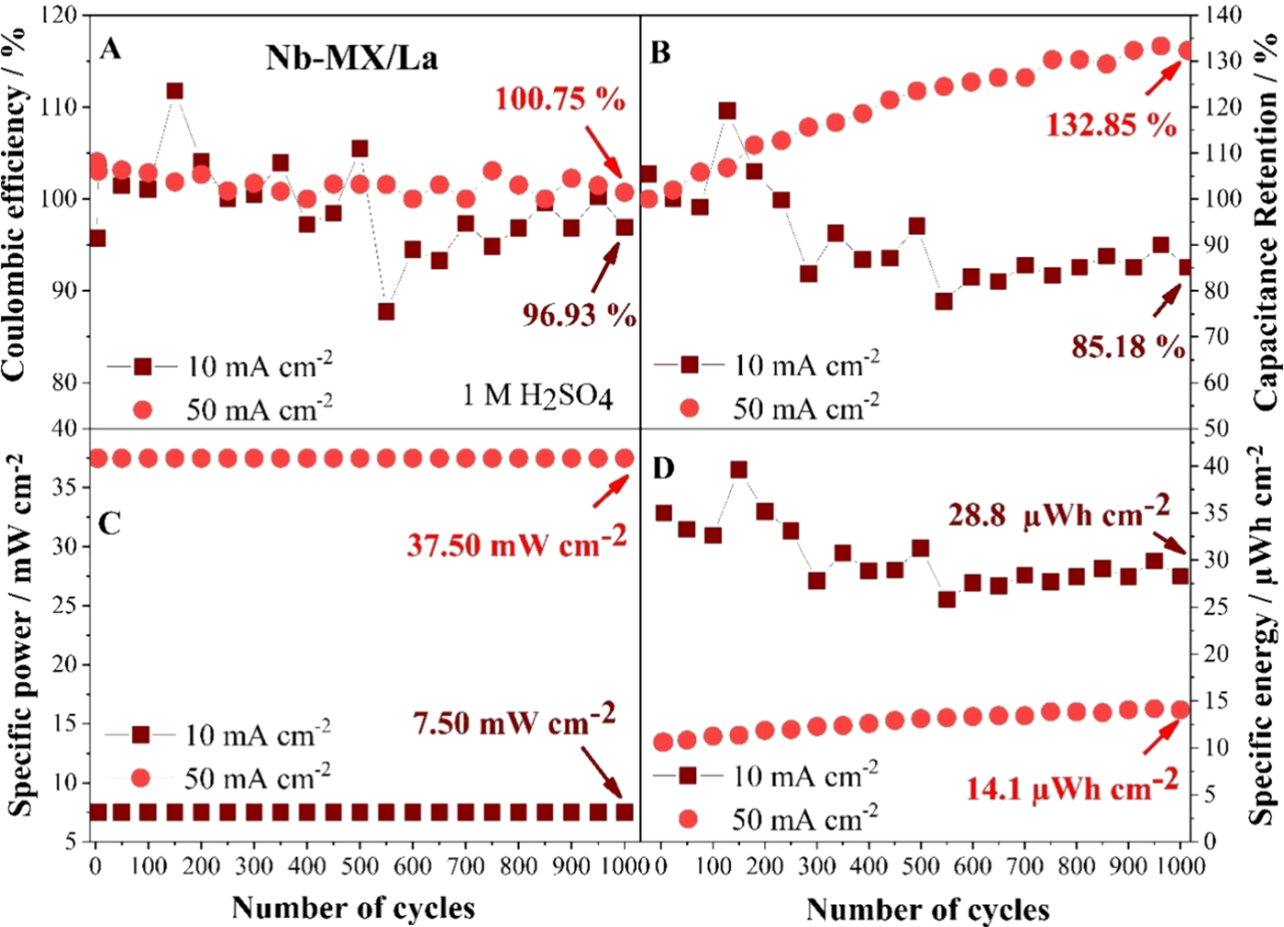
GCD performance of Nb-MX/La in 1 M H_2_SO_4_: (A) Coulombic efficiency; (B) capacitance retention; (C) specific power; and (D) specific energy.

GCD tests were performed at different current densities (Figure 7) and the electrochemical performance of Nb-MX/La and Nb-MX when subjected to lower and higher currents was compared (Figures 8, 9). The potential difference used in the CV test was the same as that used for the GCD test. However, the current densities utilized for the GCD test were lower for Nb-MX. This was due to the poor performance of the La-free material in cycles with higher currents. Notably, the performance for Nb-MX/La at higher current densities was better in terms of its Coulombic efficiency, which is characteristic of stable capacitive charge storage behavior.^55^ The capacitance retention was high, even increasing over cycles, and the specific power was high, as made evident by the charge-transfer capacity. Similar behavior was demonstrated by Nb-MX though at a lower performance compared with Nb-MX/La, showing once again that the addition of lanthanum was fundamental in the improvement of storage and transport of charges. In addition, Nb-MX/La showed an increase in charge retention over these 1000 cycles, an unusual behavior for many energy storage systems. However, as discussed in section 3.2, Figure 6D, the electrochemical behavior of the material when La is added in MXene changes with more pronounced faradaic. The material changes effects described in section 3.1, Table 1, Figure 4, such as the increased spacing between layers, improving the interface between the electrolyte and the surface of the material, the different functional groups and the change in polaron concentration enabled an increase in the availability of charge-carrying sites so that there is better performance during cycling.

In general, MXenes in aqueous electrolytes present more capacitive behavior, with low energy density and capacity; however, stored energy tends to increase when MXene presents more pronounced redox behavior. With a larger potential window, higher voltage provides a stronger interaction between the electrolyte ions and the active sites of Nb in MXene, resulting in distinct electrochemical behaviors.^45^ Thus, significant improvements in energy density were observed in Nb-MX/La compared to Nb-MX.

**Figure 10:**
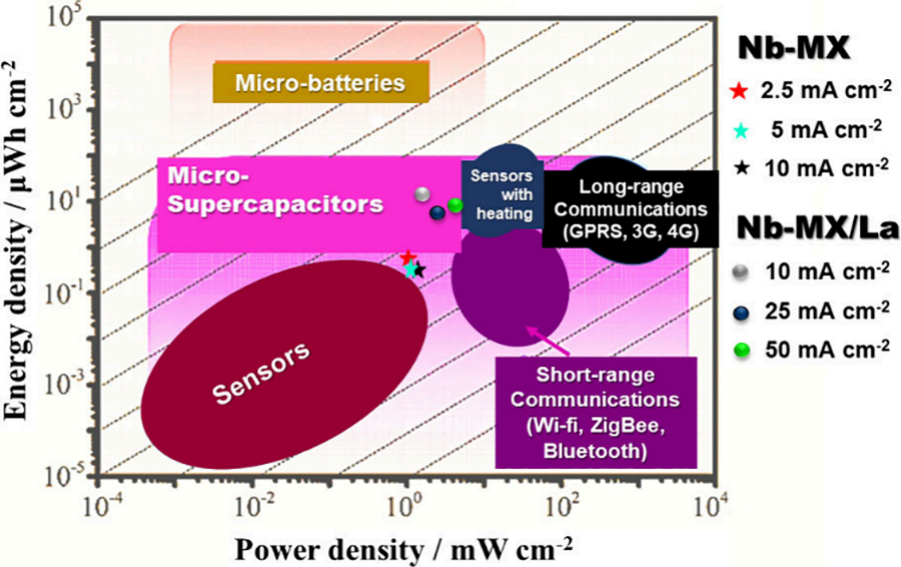
Adapted Ragone diagram^56^ indicating the performance of Nb-MX and Nb-MX/La as materials with properties for use in microsupercapacitors.

The materials were comparatively arranged on the Ragone diagram based on their specific power and energy. Although the lanthanum MXene showed better performance, both materials can be classified as having properties for future applications as active materials in microsupercapacitor devices (Figure 10).

In order to better elucidate the electrochemical properties of Nb-MX with and without La for application in energy storage, the performance of the materials obtained in this work was compared to that of different MXenes reported in the literature, Table 2.

**Table 2:** Comparison of the GCD Performance of Different MXenes for Application in Microsupercapacitors

Material	Specific Capacitance/mF cm^–2^	Specific Power/mW cm^–2^	Specific energy/μWh cm^–2^	Electrolyte	Δ*E*/V
**Nb-MXene/La**^**THIS WORK**^	**112**	**7.5**	**28.8**	**1 M H**_**2**_**SO**_**4**_	**1.5**
**Nb-MXene**^**THIS WORK**^	**10**	**5.0**	**1.45**	**1 M H**_**2**_**SO**_**4**_	**1**
Ti_3_C_2_T_*x*_^57^	7500	0.38	0.66	1 M H_2_SO_4_	0.6
Ti_3_C_2_T_*x*_^58^	25	46.6	0.77	1 M H_2_SO_4_/PVA	0.6
Ti_3_C_2_T_*x*_^59^	158	0.78	1.32	3 M H_2_SO_4_/PVA	0.6
AC/CNT/Ti_3_C_2_T_*x*_-N/GO^60^	70.1	0.83	0.42	PVA/H_2_SO_4_	0.6
PET@Ti_3_C_2_T_*x*_ NCY^61^	_	0.39	0.38	PVA/H_2_SO_4_	0.6
Ti_3_C_2_T_*x*_^62^	15.03	2.94	0.25	3 M H_2_SO_4_	0.6
Nb_2_C–AQS^63^	36	_	_	0.1 M Na_2_SO_4_	2

**Figure 11:**
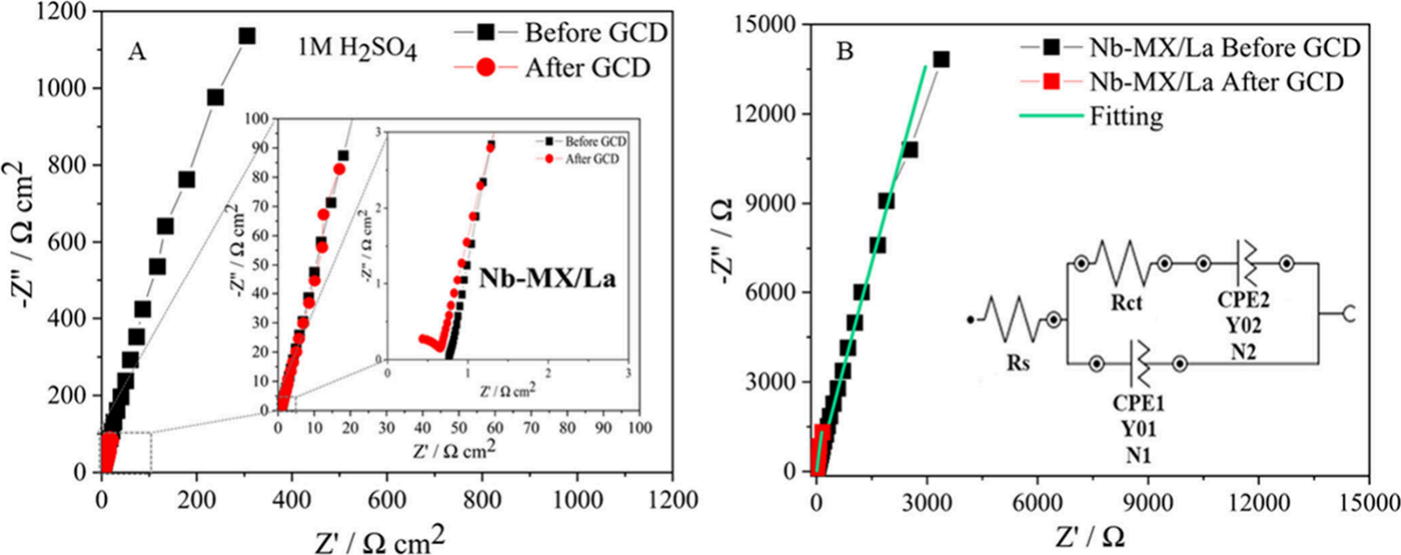
EIS and EEC analysis for Nb-MX/La comparing performance before and after 1000 GCD cycles at 50 mA cm^–2^.

**Figure 12:**
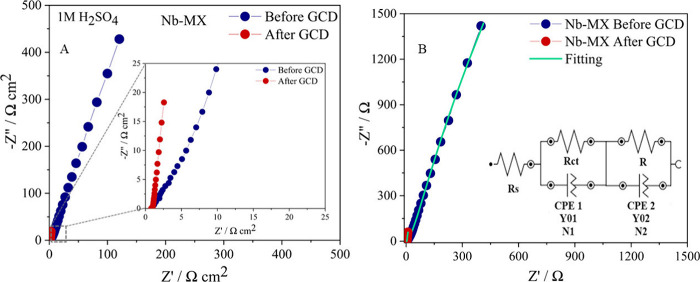
EIS and EEC analysis for Nb-MX comparing performance before and after 1000 GCD cycles at 50 mA cm^–2^.

The Nb-MX with La presented in this work exhibits the best performance over a greater range of applied potential. The specific power is only inferior to the titanium (Ti) MXene reported in the work of Kurra et al.^58^ Therefore, the insertion of La in Nb-MX favored the performance in relation to Nb-MX as well as other Ti-MXene. The EIS study was carried out before and after the GCD test at the highest current density of 10 mA cm^–2^ for Nb-MX and 50 mA cm^–2^ for Nb-MX/La and their respective electrochemical equivalent circuits (EEC) (Figures 11, 12) correlated to EIS by Complex Nonlinear Least Square analysis (Tables 3, 4).^64,65^ The capacitive behavior of the materials is improved after cycling, a phenomenon also highlighted in the work by Zhang et al.^55^ For Nb-MX/La, an increase in capacitance retention over the charge and discharge cycles is evident. This behavior is also highlighted by the values of the *N* exponent of the *CPE* being closer to 1 (Table 3) after cycling, showing that the material is more (pseudo-) capacitive, indicating more pronounced faradaic effects as a second charge storage mechanism.^12^

**Table 3:** Comparison of EEC Circuit Elements from EIS Analysis of Nb-MX/La before and after GCD Cycling at 50 mA cm^–2^^a^

Circuit elements	Nb-MX/La Before GCD	Nb-MX/La After GCD
*R*_*s*_ (Ω cm^–2^)	0.762	0.662
*R*_*ct*_ (Ω cm^–2^)	243.57	0.243
*Y*_*0*_^*CPE 1*^(Ω^–1^ s^n^) cm^–2^	9.53 × 10^–7^	4.45 × 10^–7^
*N1*	0.880	0.981
*Y*_*0*_^*CPE2*^ (Ω^–1^ s^n^) cm^–2^	1.0 × 10^–7^	3.40 × 10^–7^
*N2*	0.623	0.798
χ^2^	9.12 × 10^–3^	1.10 × 10^–2^

a χ^2^, coherence of the mathematical simulation with the experimental data; *R*_*s*_, solution resistance, *R*_*ct*_, charge transfer resistance, *Y*_*0*_^*CPE*^, admitance, *N*, CPE coefficient.

**Table 4:** Comparison of EEC Circuit Elements from EIS Analysis of Nb-MX before and after GCD Cycling at 50 mA cm^–2^^a^

Circuit elements	Nb-MX Before GCD	Nb-MX After GCD
*R*_*s*_ (Ω cm^–2^)	0.268	0.244
*R*_*ct*_ (Ω cm^–2^)	102.37	2.99
*R* (Ω cm^–2^)	0.023	0.967
*Y*_*0*_^*CPE 1*^ (Ω^–1^ s^n^) cm^–2^	1.48 × 10^–3^	1.79 × 10^–4^
N1	0.946	0.661
*Y*_*0*_^*CPE2*^ (Ω^–1^ s^n^) cm^–2^	6.80 × 10^–3^	5.65 × 10^–5^
N2	0.899	0.9
χ^2^	8.77 × 10^–3^	2.54 × 10^–2^

a χ^2^, coherence of the mathematical simulation with the experimental data; *R*_*s*_, solution resistance, *R*_*ct*_, charge transfer resistance, *R*, resistance, *Y*_*0*_^*CPE*^, admitance, *N*, CPE coefficient

The *Y*_*0*_ admittance related to charge transfer has a slight increase for Nb-MX/La after GCD. In contrast, Nb-MX, despite its more diffusive behavior with *N* closer to 0.5,^12^ has a decreasing in charge transfer after the 1000-cycle charging and discharging process. As discussed in section 3.1, MXene in an aqueous medium tends to restack itself, reducing the spacing between the layers, which limits the area of the material interacting with the ions in the electrolyte. La added to MXene acted by exchanging charges with the electrolyte and contributing to not reducing the spacing between the layers as demonstrated by its lattice parameter with a slight increase in section 3.1 and Table 1, allowing a better contact surface between the material and the electrolyte, thus presenting better charge transfer performance Inserting a heteroatom alters the properties of MXenes, i.e., electronic conductivity increases and defects are generated, which contribute to a more porous or rough texture of the material (as observed in Figure 2E), facilitating the transport of ions from the electrolyte in the MXene layers.^10^

Yu et al. inserted La combined with other metals into the MXene structure and observed an improvement in the electrochemical performance related to electrocatalysis.^66^ The high performance of MXene with La was attributed to La preventing the aggregation of layers during electrode fabrication. The synergistic effect between La and the MXene structure prevents the stacking of layers, increases the electron density facilitating the transport and storage of charges, increases the number of active sites and their accessibility to the electrolyte ions.^66^ As a result, the entire electrochemical performance of Nb-MXene/La was improved.

Still related to the parameters measured by EIS to Nb-MXene/La, there is a 3.4 times increase in *Y*_*02*_ synchronized with an increase in *N2* from 0.623 to 0.798, indicating a simultaneous decrease in diffusion transport. These two phenomena combined suggest that internal charge transfer in the electrode no longer occurs through diffusion but through a conductive phenomenon. In this case, the possibilities of restacking or pore closure are significantly reduced due to the presence of lanthanum, which was crucial in increasing the spacing between layers and stabilizing the structure, thereby improving the interaction between the electrolyte and the material compared with MXene without La. The decrease in diffusive contribution and increase in charge transfer in the material may be associated with a conduction mechanism different from diffusive, possibly due to an increase in the amount of charge carriers introduced by La. This is consistent with the increased contact between the material and the electrolyte during cycling. This is also consistent with the material changes described in the CV analysis by Dunn et al. (Figure 6). Other phenomena, such as a decrease in the resistance to charge transfer *R*_*ct*_ and the resistance of the solution *R*_*s*_ after GCD, were observed in both materials, but to a greater extent in Nb-MX/La, with *R*_*ct*_ 1000 times lower after cycling. The decrease in *R*_*ct*_ and increase in admittance in Nb-MX/La favored higher performance for specific power compared to Nb-MX, noted in the GCD test.

**Figure 13:**
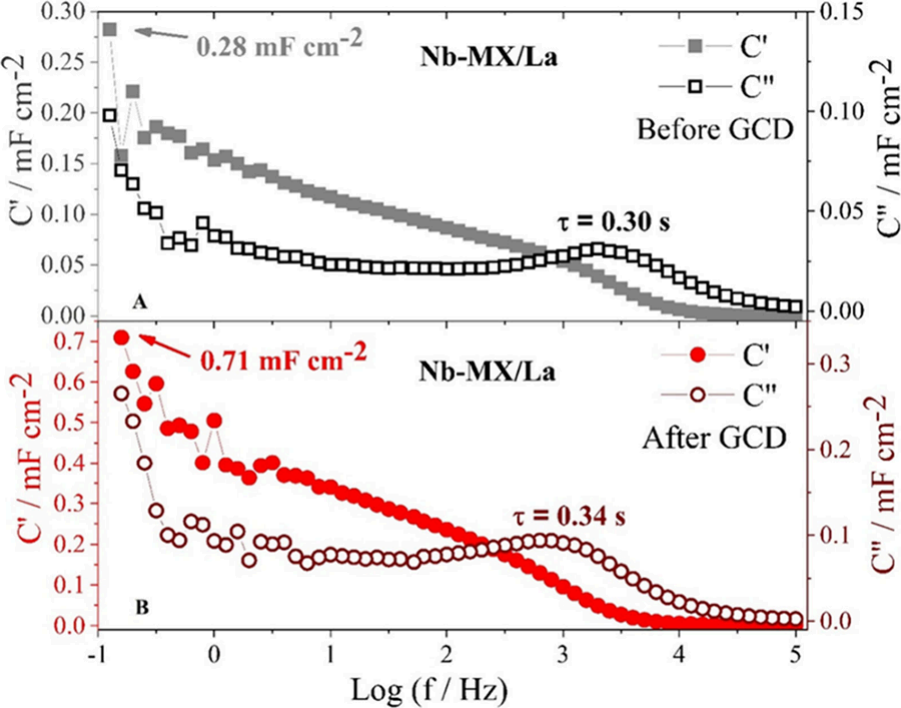
Complex capacitance and relaxation time for Nb-MX/La before and after 1000 GCD cycles at 50 mA cm^–2^.

**Figure 14:**
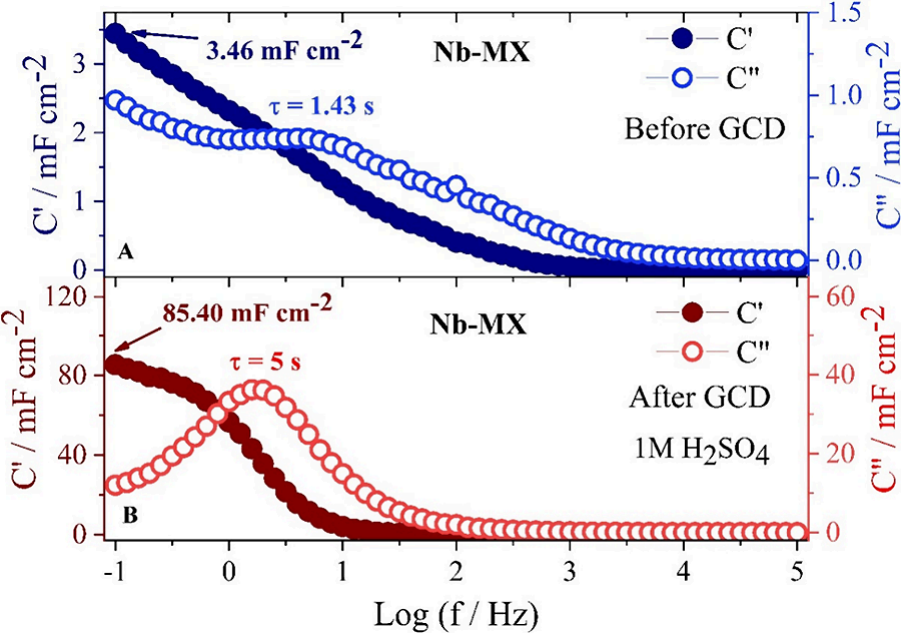
Complex capacitance and relaxation time for Nb-MX before and after 1000 GCD cycles at 50 mA cm^–2^.

The Nb-MX/La complex capacitance analysis shows lower capacitance *C’* values (Figure 13) related to the storage of charges in the cell and a shorter relaxation time, which indicates that the presence of La makes MXene more conductive with a higher charge transfer capacity. Nb-MX has a high capacitance during the discharge process along with a much longer relaxation time (Figure 14), which means that the charging and discharging process is slower, and there is more charge storage in the cell. However, even with greater charge storage at low frequencies, Nb-MX/La performed better in the energy density GCD test. This is because, despite storing more charges with high *C’* values, Nb-MX has a high *C″* value at low frequencies, which means irreversible energy dissipation and charges not effectively used by the device.

## 4 Conclusion

The synthesis of Nb-MX and the process of La insertion into its structure (Nb-MX/La) were successful. The addition of La to the Nb-MX was crucial in stabilizing the material’s structure by increasing the spacing between layers, improving interaction with the electrolyte, and enabling charge/discharge cycling in wider potential windows and at higher current density in an aqueous medium. Nb-MX/La achieved a specific capacitance of up to 157 mF cm^–2^, a specific capacity of 42 mAh cm^–2^ at 250 mV s^–1^, and a specific power of 37.5 mW cm^–2^, a specific energy of 14.1 mWh cm^–2^ after 1000 charge and discharge cycles at 50 mA cm^–2^. Therefore, Nb-MX/La is a promising material with high electrochemical performance that can be applied as an active electrode mass in energy storage devices such as aqueous microsupercapacitors.

## Data Availability

The data that support the tables within this paper and other findings of this study are available from the corresponding author upon reasonable request.

## References

[ref1] Boonpakdee D.; Guajardo Yévenes C. F.; Surareungchai W.; La-o-vorakiat C. Exploring Non-Linearities of Carbon-Based Microsupercapacitors from an Equivalent Circuit Perspective. J. Mater. Chem. A 2018, 6 (16), 7162–7167. 10.1039/C8TA01995A.

[ref2] Bu F.; Zhou W.; Xu Y.; Du Y.; Guan C.; Huang W. Recent Developments of Advanced Micro-Supercapacitors: Design, Fabrication and Applications. npj Flexible Electronics 2020, 4 (1), 31 10.1038/s41528-020-00093-6.

[ref3] Downes M.; Shuck C. E.; McBride B.; Busa J.; Gogotsi Y. Comprehensive Synthesis of Ti3C2Tx from MAX Phase to MXene. Nat. Protoc. 2024, 19, 1807 10.1038/s41596-024-00969-1. 38504139

[ref4] Zhu Y.; Wang S.; Ma J.; Das P.; Zheng S.; Wu Z.-S. Recent Status and Future Perspectives of 2D MXene for Micro-Supercapacitors and Micro-Batteries. Energy Storage Materials 2022, 51, 500–526. 10.1016/j.ensm.2022.06.044.

[ref5] Zhang C.; McKeon L.; Kremer M. P.; Park S.-H.; Ronan O.; Seral-Ascaso A.; Barwich S.; Coileáin C. Ó.; McEvoy N.; Nerl H. C.; Anasori B.; Coleman J. N.; Gogotsi Y.; Nicolosi V. Additive-Free MXene Inks and Direct Printing of Micro-Supercapacitors. Nat. Commun. 2019, 10 (1), 1795 10.1038/s41467-019-09398-1. 30996224 PMC6470171

[ref6] Li X.; Ma Y.; Shen P.; Zhang C.; Cao M.; Xiao S.; Yan J.; Luo S.; Gao Y. An Ultrahigh Energy Density Flexible Asymmetric Microsupercapacitor Based on Ti3C2Tx and PPy/MnO2 with Wide Voltage Window. Advanced Materials Technologies 2020, 5 (8), 2000272 10.1002/admt.202000272.

[ref7] Zhu Y.; Zhang Q.; Ma J.; Das P.; Zhang L.; Liu H.; Wang S.; Li H.; Wu Z.-S. Three-Dimensional (3D)-Printed MXene High-Voltage Aqueous Micro-Supercapacitors with Ultrahigh Areal Energy Density and Low-Temperature Tolerance. Carbon Energy 2024, 6, e481 10.1002/cey2.481.

[ref8] Zhang C. J.; Pinilla S.; McEvoy N.; Cullen C. P.; Anasori B.; Long E.; Park S.-H.; Seral-Ascaso A.; Shmeliov A.; Krishnan D.; Morant C.; Liu X.; Duesberg G. S.; Gogotsi Y.; Nicolosi V. Oxidation Stability of Colloidal Two-Dimensional Titanium Carbides (MXenes). Chem. Mater. 2017, 29 (11), 4848–4856. 10.1021/acs.chemmater.7b00745.

[ref9] Wang X.; Sun G.; Routh P.; Kim D.-H.; Huang W.; Chen P. Heteroatom-Doped Graphene Materials: Syntheses, Properties and Applications. Chem. Soc. Rev. 2014, 43 (20), 7067–7098. 10.1039/C4CS00141A. 24954470

[ref10] Dey A.; Varagnolo S.; Power N. P.; Vangapally N.; Elias Y.; Damptey L.; Jaato B. N.; Gopalan S.; Golrokhi Z.; Sonar P.; Selvaraj V.; Aurbach D.; Krishnamurthy S. Doped MXenes—A New Paradigm in 2D Systems: Synthesis, Properties and Applications. Prog. Mater. Sci. 2023, 139, 101166 10.1016/j.pmatsci.2023.101166.

[ref11] Gandara M.; Gonçalves E. S. Electroactive Composites: PANI Electrochemical Synthesis with GO and rGO for Structural Carbon Fiber Coating. Prog. Org. Coat. 2020, 138, 105399 10.1016/j.porgcoat.2019.105399.

[ref12] Gandara M.; Gonçalves E. S. Polyaniline Supercapacitor Electrode and Carbon Fiber Graphene Oxide: Electroactive Properties at the Charging Limit. Electrochim. Acta 2020, 345, 136197 10.1016/j.electacta.2020.136197.

[ref13] Sarkar S.; Akshaya R.; Ghosh S. Nitrogen Doped Graphene/CuCr2O4 Nanocomposites for Supercapacitors Application: Effect of Nitrogen Doping on Coulombic Efficiency. Electrochim. Acta 2020, 332, 135368 10.1016/j.electacta.2019.135368.

[ref14] Rajeevan S.; John S.; Ponnamma D.; George S. C. Fabrication of High-Performance Symmetric Supercapacitor of Graphene Electrodes by Tuning Their Electrochemical Properties. Journal of Energy Storage 2022, 56, 105919 10.1016/j.est.2022.105919.

[ref15] Taberna P. L.; Simon P.; Fauvarque J. F. Electrochemical Characteristics and Impedance Spectroscopy Studies of Carbon-Carbon Supercapacitors. J. Electrochem. Soc. 2003, 150 (3), A292 10.1149/1.1543948.

[ref16] Almeida D. A. L.; Couto A. B.; Ferreira N. G. Flexible Polyaniline/Reduced Graphene Oxide/Carbon Fiber Composites Applied as Electrodes for Supercapacitors. J. Alloys Compd. 2019, 788, 453–460. 10.1016/j.jallcom.2019.02.194.

[ref17] Yu D. S. ICDD Grant-in-Aid, 1975.

[ref18] Hu C.; Li F.; Zhang J.; Wang J.; Wang J.; Zhou Y. Nb4AlC3: A New Compound Belonging to the MAX Phases. Scripta Materialia 2007, 57 (10), 893–896. 10.1016/j.scriptamat.2007.07.038.

[ref19] Zhou J.; Tao Q.; Ahmed B.; Palisaitis J.; Persson I.; Halim J.; Barsoum M. W.; Persson P. O. Å.; Rosen J. High-Entropy Laminate Metal Carbide (MAX Phase) and Its Two-Dimensional Derivative MXene. Chem. Mater. 2022, 34 (5), 2098–2106. 10.1021/acs.chemmater.1c03348.

[ref20] Ma X.; Wang A.; Miao J.; Fan T. 2D Lamellar Membrane with MXene Hetero-Intercalated Small Sized Graphene Oxide for Harsh Environmental Wastewater Treatment. Sep. Purif. Technol. 2023, 311, 123248 10.1016/j.seppur.2023.123248.

[ref21] Shekhirev M.; Shuck C. E.; Sarycheva A.; Gogotsi Y. Characterization of MXenes at Every Step, from Their Precursors to Single Flakes and Assembled Films. Prog. Mater. Sci. 2021, 120, 100757 10.1016/j.pmatsci.2020.100757.

[ref22] Rafiq S.; Awan S.; Zheng R.-K.; Wen Z.; Rani M.; Akinwande D.; Rizwan S. Novel Room-Temperature Ferromagnetism in Gd-Doped 2-Dimensional Ti3C2Tx MXene Semiconductor for Spintronics. J. Magn. Magn. Mater. 2020, 497, 165954 10.1016/j.jmmm.2019.165954.

[ref23] Zhao S.; Wang X.; Kurra N.; Gogotsi Y.; Gao Y. Effect of Pinholes in Nb4C3MXene Sheets on Its Electrochemical Behavior in Aqueous Electrolytes. Electrochem. Commun. 2022, 142, 107380 10.1016/j.elecom.2022.107380.

[ref24] Yu H.; Wang Y.; Jing Y.; Ma J.; Du C.-F.; Yan Q. Surface Modified MXene-Based Nanocomposites for Electrochemical Energy Conversion and Storage. Small 2019, 15 (25), 1901503 10.1002/smll.201901503. 31066206

[ref25] Yang Y.; Anayee M.; Pattammattel A.; Shekhirev M.; Wang R.; Huang X.; Chu Y. S.; Gogotsi Y.; May S. J. Enhanced Magnetic Susceptibility in Ti3C2Tx MXene with Co and Ni Incorporation. Nanoscale 2024, 16 (11), 5760–5767. 10.1039/D3NR05685F. 38412012

[ref26] Iqbal M.; Fatheema J.; Noor Q.; Rani M.; Mumtaz M.; Zheng R.-K.; Khan S. A.; Rizwan S. Co-Existence of Magnetic Phases in Two-Dimensional MXene. Materials Today Chemistry 2020, 16, 100271 10.1016/j.mtchem.2020.100271.

[ref27] Zhang Q.; Zhou J.; Zeng G.; Ren S. Effect of Lanthanum and Yttrium Doped LiFePO4 Cathodes on Electrochemical Performance of Lithium-Ion Battery. J. Mater. Sci. 2023, 58 (20), 8463–8477. 10.1007/s10853-023-08542-z.

[ref28] Farid G.; Murtaza G.; Umair M.; Arif H. S.; Ali H. S.; Muhammad N.; Ahmad M. Effect of La-Doping on the Structural, Morphological and Electrochemical Properties of LiCoO2 Nanoparticles Using Sol-Gel Technique. Materials Research Express 2018, 5 (5), 055044 10.1088/2053-1591/aac4dc.

[ref29] Zaheer A.; Zahra S. A.; Iqbal M. Z.; Mahmood A.; Khan S. A.; Rizwan S. Nickel-Adsorbed Two-Dimensional Nb2C MXene for Enhanced Energy Storage Applications. RSC Adv. 2022, 12 (8), 4624–4634. 10.1039/D2RA00014H. 35425492 PMC8981252

[ref30] Ni Y.; Yang J.; Sun L.; Liu Q.; Fei Z.; Chen X.; Zhang Z.; Tang J.; Cui M.; Qiao X. La/LaF3 Co-Modified MIL-53(Cr) as an Efficient Adsorbent for the Removal of Tetracycline. Journal of Hazardous Materials 2022, 426, 128112 10.1016/j.jhazmat.2021.128112. 34965495

[ref31] Sunding M. F.; Hadidi K.; Diplas S.; Lo̷vvik O. M.; Norby T. E.; Gunnæs A. E. XPS Characterisation of in Situ Treated Lanthanum Oxide and Hydroxide Using Tailored Charge Referencing and Peak Fitting Procedures. J. Electron Spectrosc. Relat. Phenom. 2011, 184 (7), 399–409. 10.1016/j.elspec.2011.04.002.

[ref32] Seo J.; Jeong S.; Kim S. Visible-Light-Driven Water Splitting over Particulate LaNbON2 Prepared from La-Rich Lanthanum Niobium Oxides. ACS Appl. Energy Mater. 2021, 4 (4), 3141–3150. 10.1021/acsaem.0c02930.

[ref33] Lee J.-W.; Jeong S.-P.; You N.-H.; Moon S.-Y. Tunable Synthesis of Predominant Semi-Ionic and Covalent Fluorine Bonding States on a Graphene Surface. Nanomaterials 2021, 11 (4), 942 10.3390/nano11040942. 33917149 PMC8067876

[ref34] Cai P.; He Q.; Wang L.; Liu X.; Yin J.; Liu Y.; Huang Y.; Huang Z. Two-Dimensional Nb-Based M4C3Tx MXenes and Their Sodium Storage Performances. Ceram. Int. 2019, 45 (5), 5761–5767. 10.1016/j.ceramint.2018.12.042.

[ref35] Fatheema J.; Khan S. A.; Arif N.; Iqbal M.; Ullah H.; Rizwan S. Meissner to Ferromagnetic Phase Transition in La-Decorated Functionalized Nb2C MXene: An Experimental and Computational Analysis. Nanotechnology 2021, 32 (8), 085711 10.1088/1361-6528/abc7d3. 33152725

[ref36] Koua K. A. J.; Peng J.; Zhang P.; Li N. Unveiling the Magnetic Ordering Effect in La-Doped Ti3C2O2MXenes on Electrocatalytic CO2 Reduction. J. Mater. Chem. A 2023, 12 (1), 303–313. 10.1039/D3TA06457C.

[ref37] Bae S.; Kang Y.-G.; Khazaei M.; Ohno K.; Kim Y.-H.; Han M. J.; Chang K. J.; Raebiger H. Electronic and Magnetic Properties of Carbide MXenes—the Role of Electron Correlations. Materials Today Advances 2021, 9, 100118 10.1016/j.mtadv.2020.100118.

[ref38] Krinichnyi V. I.; Yudanova E. I.; Wessling B. Influence of Spin–Spin Exchange on Charge Transfer in PANI-ES/P3DDT/PCBM Composite. Synth. Met. 2013, 179, 67–73. 10.1016/j.synthmet.2013.07.008.

[ref39] Mišurović J.; Mojović M.; Marjanović B.; Vulić P.; Ćirić-Marjanović G. Magnetite Nanoparticles-Catalysed Synthesis of Conductive Polyaniline. Synth. Met. 2019, 257, 116174 10.1016/j.synthmet.2019.116174.

[ref40] Rao P.; Sathyanarayana D. Electron Spin Resonance Spectroscopy and Electrical Conductivity Studies on Some Polyaniline Salts and Their Bases. Indian Journal of Chemistry Section a 2004, 43, 1377–1384.

[ref41] Zheng W.; Sun B.; Li D.; Gali S. M.; Zhang H.; Fu S.; Di Virgilio L.; Li Z.; Yang S.; Zhou S.; Beljonne D.; Yu M.; Feng X.; Wang H. I.; Bonn M. Band Transport by Large Fröhlich Polarons in MXenes. Nat. Phys. 2022, 18 (5), 544–550. 10.1038/s41567-022-01541-y.

[ref42] Palagonia M. S.; Erinmwingbovo C.; Brogioli D.; Mantia F. L. Comparison between Cyclic Voltammetry and Differential Charge Plots from Galvanostatic Cycling. J. Electroanal. Chem. 2019, 847, 113170 10.1016/j.jelechem.2019.05.052.

[ref43] Zhang S.; Pan N. Supercapacitors Performance Evaluation. Adv. Energy Mater. 2015, 5 (6), 1401401 10.1002/aenm.201401401.

[ref44] Brousse T.; Bélanger D.; Long J. W. To Be or Not To Be Pseudocapacitive?. J. Electrochem. Soc. 2015, 162 (5), A5185 10.1149/2.0201505jes.

[ref45] Li X.; Ma X.; Hou Y.; Zhang Z.; Lu Y.; Huang Z.; Liang G.; Li M.; Yang Q.; Ma J.; Li N.; Dong B.; Huang Q.; Chen F.; Fan J.; Zhi C. Intrinsic Voltage Plateau of a Nb2CTx MXene Cathode in an Aqueous Electrolyte Induced by High-Voltage Scanning. Joule 2021, 5 (11), 2993–3005. 10.1016/j.joule.2021.09.006.

[ref46] Huang X.; Wu P. A Facile, High-Yield, and Freeze-and-Thaw-Assisted Approach to Fabricate MXene with Plentiful Wrinkles and Its Application in On-Chip Micro-Supercapacitors. Adv. Funct. Mater. 2020, 30 (12), 1910048 10.1002/adfm.201910048.

[ref47] Yue Y.; Liu N.; Ma Y.; Wang S.; Liu W.; Luo C.; Zhang H.; Cheng F.; Rao J.; Hu X.; Su J.; Gao Y. Highly Self-Healable 3D Microsupercapacitor with MXene–Graphene Composite Aerogel. ACS Nano 2018, 12 (5), 4224–4232. 10.1021/acsnano.7b07528. 29648800

[ref48] Wang G.; Zhang R.; Zhang H.; Cheng K. Aqueous MXene Inks for Inkjet-Printing Microsupercapacitors with Ultrahigh Energy Densities. J. Colloid Interface Sci. 2023, 645, 359–370. 10.1016/j.jcis.2023.04.155. 37156144

[ref49] Cheng W.; Fu J.; Hu H.; Ho D. Interlayer Structure Engineering of MXene-Based Capacitor-Type Electrode for Hybrid Micro-Supercapacitor toward Battery-Level Energy Density. Advanced Science 2021, 8 (16), 2100775 10.1002/advs.202100775. 34137521 PMC8373094

[ref50] Zhao R.; Elzatahry A.; Chao D.; Zhao D. Making MXenes More Energetic in Aqueous Battery. Matter 2022, 5 (1), 8–10. 10.1016/j.matt.2021.12.005.

[ref51] Mathis T. S.; Kurra N.; Wang X.; Pinto D.; Simon P.; Gogotsi Y. Energy Storage Data Reporting in Perspective—Guidelines for Interpreting the Performance of Electrochemical Energy Storage Systems. Adv. Energy Mater. 2019, 9 (39), 1902007 10.1002/aenm.201902007.

[ref52] Huang H.; Cui J.; Liu G.; Bi R.; Zhang L. Carbon-Coated MoSe2/MXene Hybrid Nanosheets for Superior Potassium Storage. ACS Nano 2019, 13 (3), 3448–3456. 10.1021/acsnano.8b09548. 30817126

[ref53] Guan Y.; Zhao R.; Cong Y.; Chen K.; Wu J.; Zhu H.; Dong Z.; Zhang Q.; Yuan G.; Li Y.; Zhang J.; Li X. Flexible Ti2C MXene Film: Synthesis, Electrochemical Performance and Capacitance Behavior. Chemical Engineering Journal 2022, 433, 133582 10.1016/j.cej.2021.133582.

[ref54] Kawai K.; Fujita M.; Iizuka R.; Yamada A.; Okubo M. Influence of Surface Termination Groups on Electrochemical Charge Storage of MXene Electrodes. 2D Materials 2023, 10 (1), 014012 10.1088/2053-1583/aca1cf.

[ref55] Zhang C.; Kremer M. P.; Seral-Ascaso A.; Park S.-H.; McEvoy N.; Anasori B.; Gogotsi Y.; Nicolosi V. Stamping of Flexible, Coplanar Micro-Supercapacitors Using MXene Inks. Adv. Funct. Mater. 2018, 28 (9), 1705506 10.1002/adfm.201705506.

[ref56] Liu L.; Zhao H.; Lei Y. Advances on Three-Dimensional Electrodes for Micro-Supercapacitors: A Mini-Review. InfoMat 2019, 1 (1), 74–84. 10.1002/inf2.12007.

[ref57] Yang C.; Wu X.; Xia H.; Zhou J.; Wu Y.; Yang R.; Zhou G.; Qiu L. 3D Printed Template-Assisted Assembly of Additive-Free Ti3C2Tx MXene Microlattices with Customized Structures toward High Areal Capacitance. ACS Nano 2022, 16 (2), 2699–2710. 10.1021/acsnano.1c09622. 35084815

[ref58] Kurra N.; Ahmed B.; Gogotsi Y.; Alshareef H. N. MXene-on-Paper Coplanar Microsupercapacitors. Adv. Energy Mater. 2016, 6 (24), 1601372 10.1002/aenm.201601372.

[ref59] Abdolhosseinzadeh S.; Schneider R.; Verma A.; Heier J.; Nüesch F.; Zhang C. Turning Trash into Treasure: Additive Free MXene Sediment Inks for Screen-Printed Micro-Supercapacitors. Adv. Mater. 2020, 32 (17), 2000716 10.1002/adma.202000716. 32196130

[ref60] Yu L.; Fan Z.; Shao Y.; Tian Z.; Sun J.; Liu Z. Versatile N-Doped MXene Ink for Printed Electrochemical Energy Storage Application. Adv. Energy Mater. 2019, 9 (34), 1901839 10.1002/aenm.201901839.

[ref61] Shao W.; Tebyetekerwa M.; Marriam I.; Li W.; Wu Y.; Peng S.; Ramakrishna S.; Yang S.; Zhu M. Polyester@MXene Nanofibers-Based Yarn Electrodes. J. Power Sources 2018, 396, 683–690. 10.1016/j.jpowsour.2018.06.084.

[ref62] Tang J.; Yi W.; Zhong X.; Zhang C.; Xiao X.; Pan F.; Xu B. Laser Writing of the Restacked Titanium Carbide MXene for High Performance Supercapacitors. Energy Storage Materials 2020, 32, 418–424. 10.1016/j.ensm.2020.07.028.

[ref63] Wang G.; Yang Z.; Nie X.; Wang M.; Liu X. A Flexible Supercapacitor Based on Niobium Carbide MXene and Sodium Anthraquinone-2-Sulfonate Composite Electrode. Micromachines 2023, 14 (8), 1515 10.3390/mi14081515. 37630052 PMC10456233

[ref64] Boukamp B. A. A Nonlinear Least Squares Fit Procedure for Analysis of Immittance Data of Electrochemical Systems. Solid State Ionics 1986, 20 (1), 31–44. 10.1016/0167-2738(86)90031-7.

[ref65] Boukamp B. A. A Package for Impedance/Admittance Data Analysis. Solid State Ionics 1986, 18–19, 136–140. 10.1016/0167-2738(86)90100-1.

[ref66] Yu M.; Zheng J.; Guo M. La-Doped NiFe-LDH Coupled with Hierarchical Vertically Aligned MXene Frameworks for Efficient Overall Water Splitting. Journal of Energy Chemistry 2022, 70, 472–479. 10.1016/j.jechem.2022.02.044.

